# Photoacoustic topography through an ergodic relay for functional imaging and biometric application *in vivo*

**DOI:** 10.1117/1.JBO.25.7.070501

**Published:** 2020-07-10

**Authors:** Yang Li, Lei Li, Liren Zhu, Junhui Shi, Konstantin Maslov, Lihong V. Wang

**Affiliations:** a California Institute of Technology, Caltech Optical Imaging Laboratory, Andrew and Peggy Cherng Department of Medical Engineering, Pasadena, California, United States; b California Institute of Technology, Department of Electrical Engineering, Pasadena, California, United States; c Washington University in St. Louis, Department of Biomedical Engineering, St. Louis, Missouri, United States

**Keywords:** photoacoustics, biomedical optics, imaging systems

## Abstract

**Significance:** Photoacoustic (PA) tomography has demonstrated versatile biomedical applications. However, an array-based PA computed tomography (PACT) system is complex and expensive, whereas a single-element detector-based scanning PA system is too slow to detect some fast biological dynamics *in vivo*. New PA imaging methods are sought after.

**Aim:** To overcome these limitations, we developed photoacoustic topography through an ergodic relay (PATER), a novel high-speed imaging system with a single-element detector.

**Approach:** PATER images widefield PA signals encoded by the acoustic ergodic relay with a single-laser shot.

**Results:** We applied PATER *in vivo* to monitor changes in oxygen saturation in a mouse brain and also to demonstrate high-speed matching of vascular patterns for biometric authentication.

**Conclusions:** PATER has achieved a high-speed temporal resolution over a large field of view. Our results suggest that PATER is a promising and economical alternative to PACT for fast imaging.

Photoacoustic (PA) imaging provides functional and molecular information by sensing optical absorption, which supports a wide range of biomedical applications.^1^^–^^4^ PA computed tomography (PACT) has successfully imaged structural and dynamic features in animals and humans.^5^^–^^7^ Using an array of ultrasonic transducers, a PACT system can detect signals from a large field of view (FOV) in parallel, but the multichannel detection and acquisition system is complex and expensive.^8^^,^^9^ Moreover, PACT systems are often bulky. On the other hand, conventional PA microscopy systems scan a single-element ultrasonic transducer to form images, with reduced imaging throughput.^4^^,^^10^

As an alternative, we developed photoacoustic topography through an ergodic relay (PATER), a novel high-speed imaging technology with a single-element ultrasonic transducer.^11^ An acoustic ergodic relay (ER) is an acoustic waveguide that encodes sound waves from the input points to an output point with distinct acoustic reverberant characteristics.^12^ We had previously shown that a right-angle prism works as an ER for PATER.^11^ Using only a single-element ultrasonic transducer, PATER simultaneously detects widefield PA signals encoded by the ER and then mathematically decodes the received signal to form a widefield image.^11^^,^^13^^,^^14^ Consequently, PATER can be used to study dynamic activities with a submillisecond temporal resolution over a large FOV. Applying PATER *in vivo*, we monitored changes in oxygen saturation in a mouse brain and demonstrated high-speed recognition of vascular patterns for biometric authentication. Our results have demonstrated that PATER is a promising and economical alternative to PACT for a broad range of biomedical applications.

Figure 1(a) shows a schematic of the PATER system. A 532-nm pulsed laser beam (INNOSAB IS8II-E, Edgewave GmbH, 5-ns pulse width, and 1-kHz pulse repetition rate) passes through an optical-element wheel (LTFW6, Thorlabs, Inc.), which switches the active optical elements (a lens and an engineered diffuser) in and out of the light path according to the acquisition mode. The laser beam then passes through the ER and illuminates the object on the ER’s imaging plane. PA waves are encoded inside the ER and finally detected by a single-element ultrasonic transducer (VP-0.5-20 MHz, CTS Electronics, Inc.).

**Fig. 1:**
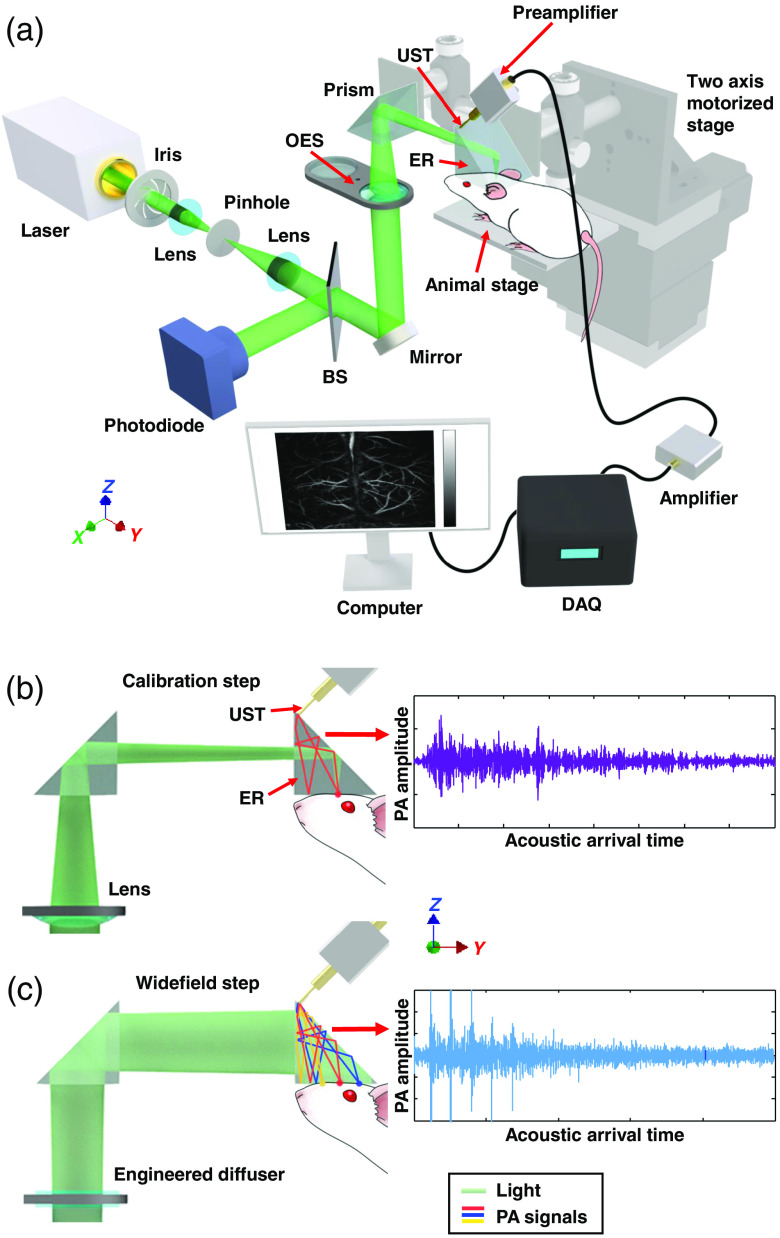
PATER: (a) schematic of the system. BS, beam splitter; DAQ, data-acquisition unit; ER, ergodic relay; OEW, optical-element wheel; and UST, ultrasonic transducer. The OEW switches the two optical elements (a lens or an engineered diffuser) according to the acquisition mode (calibration or widefield). (b) Schematic for the calibration mode. The light is focused by a lens to generate PA waves. Each detected PA signal represents the impulse response from the focused illumination position. (c) Schematic for the widefield imaging mode. The widefield light is homogenized by an engineered diffuser to illuminate the entire FOV homogeneously. The detected PA signal is a linear combination of impulse responses from the entire FOV.

PATER requires two acquisition steps. In the first step—a point-by-point scanning called calibration mode [Fig. 1(b)], a laser beam is focused by a plano-convex lens (LA1433, Thorlabs, Inc.; 150-mm focal length) to a small spot (∼30  μm) on the input surface of the ER that interfaces with the object to be imaged. Because the pulse width of the laser (∼5  ns) is much shorter than the central period of the ultrasonic transducer (50 ns, correspoinding to 20 MHz) and the focused beam spot (∼30  μm) is much smaller than the central acoustic wavelength (∼300  μm inside the ER), each PA wave input to the ER can be approximated as a spatiotemporal delta function.^11^^,^^15^ Therefore, each calibration measurement quantifies the impulse response of the system at one scanning position. The ER is driven by a customized two-axis motorized stage for raster scanning along the x and y axes, so impulse responses over the entire FOV can be calibrated. The second step, referred to as widefield imaging mode, uses a broad laser beam for illumination [Fig. 1(c)]. The laser beam passes through an engineered diffuser (EDC-5-A-1r, RPC Photonics, Inc.; 5.5-deg divergence angle) that homogenizes the beam for uniform illumination.

PATER’s system setup and reconstruction method were reported in Ref. 11. Each widefield measurement can be expressed as a linear combination of the impulse responses from all pixels:

$$s(t)=\sum_{i=1}^{N_{p}}k_{i}(t)P_{i},$$ (1)

where s denotes the detected widefield PA signal, t is the time, i is the pixel index, $N_{p}$ is the total number of pixels, $k_{i}$ is the normalized impulse response from the calibration, and $P_{i}$ is the root-mean-squared (RMS) PA amplitude.^11^ The RMS value of the raw calibration signal ${\tilde{k}}_{i}(t)$ for the i’th pixel was calculated as

$${\mathrm{RMS}}_{i}=\sqrt{\frac{1}{N_{t}}\overset{N_{t}}{\underset{j=1}{\sum }}\;{\left[{\overset{˜}{k}}_{i}(t_{j})\right]}^{2}},$$ (2)

where $N_{t}$ denotes the number of sampled time points and t is the time. A 2-D density plot of ${\mathrm{RMS}}_{i}$ over all pixels is a calibration image. To construct the system matrix K, the normalized impulse response was computed for each time point through $k_{i}(t_{j})={\overset{˜}{k}}_{i}(t_{j})/{\mathrm{RMS}}_{i}$. Eq. (1) can be recast to matrix form by discretizing time t:

s=KP, (3)

where $K=[k_{1},k_{2},\ldots ,k_{N_{p}}]$ is the system matrix. Pixels with RMS values lower than twice (6 dB) the noise amplitude were considered as the background that was too dark to calibrate for; therefore, the impulse responses of these pixels were excluded from the system matrix K. The widefield image P is reconstructed by solving the inverse problem of Eq. (3) as a minimizer of the objective function, adopting a two-step iterative shrinkage/thresholding algorithm:^16^

P^=arg minP‖s−KP‖2+2λΦTV(P). (4)

Here $\Phi_{\mathrm{TV}}(P)$ is the total variation regularization term and λ is the regularization parameter.^16^

We tested the linearity of the PATER system by measuring concentrations of the Evans Blue (EB) dye (E2129, Sigma-Aldrich, Inc.) in two tubes with 532-nm light illumination. Two silicone tubes with a 0.65-mm inner diameter were placed on the ER surface in parallel, separated by ∼3  mm. Ultrasonic gel was applied between the tubes and the ER to facilitate acoustic coupling. An EB solution with a 0.6% concentration by mass was injected into the two tubes for calibration. The concentration of EB in one tube was kept unchanged as a control, whereas the concentration of EB in the other tube was varied from 0% to 0.9% [Fig. 2(a)]. The measured concentrations, calculated based on the widefield images, agreed well with the preset concentrations [Figs. 2(b) and 2(c)], which proved the linearity of the PATER system’s widefield measurement.

**Fig. 2:**
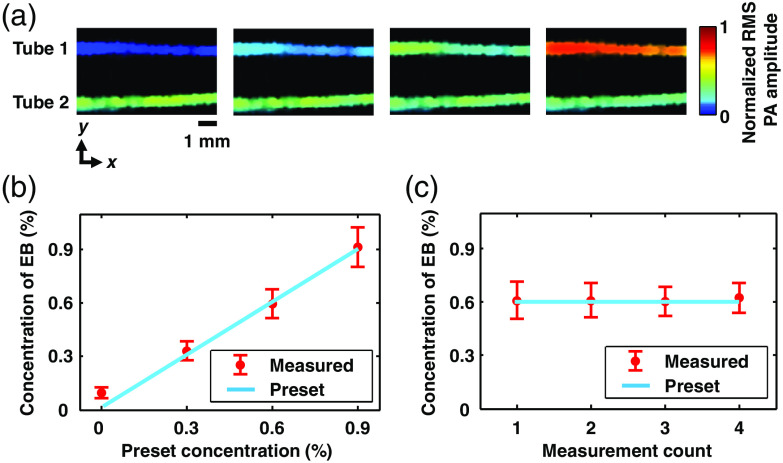
Quantification of EB dye concentration in tubes. (a) Widefield images of EB in the two tubes. Tube 2 was the control, where the EB’s concentration by mass remained at 0.6%. The concentration of EB in tube 1 varied from 0% to 0.9% from left to right, with a step size of 0.3%. (b) Concentration of EB measured by PATER versus the preset concentration of EB in tube 1. (c) Concentration of EB in tube 2 measured by PATER as the control for each measurement. Error bars, standard deviations of the pixel values within the corresponding tubes.

For *in vivo* studies, we used female ND4 Swiss Webster mice (Envigo; 18 to 20 g, 6 to 8 weeks). All the laboratory animal protocols were approved by the Animal Studies Committee of Washington University in St. Louis and the Institutional Animal Care and Use Committee of California Institute of Technology. The mouse was anesthetized in a small chamber with 5% vaporized isoflurane mixed with air for anesthesia induction and then transferred to a customized animal mount where it was kept anesthetized with a continuous supply of 1.5% vaporized isoflurane. The animal mount consisted of a stereotaxic frame that fixed the mouse’s head and a heating pad that maintained the mouse’s body temperature at ∼38°C. The hair on the mouse’s head was razor trimmed, and the scalp was surgically removed, but the skull was left intact. The scalp was removed to enable direct contact between the skull and ER to facilitate acoustic coupling. Bloodstains on the skull were carefully cleaned off with phosphate buffered saline solution, and ultrasound gel was applied on the skull for acoustic coupling. Then the animal mount was raised until the mouse’s skull was in contact with the imaging surface of the ER. An adequate amount of pressure was maintained between the mounted animal and the ER to prevent the mouse’s head from moving, but not so much pressure as to interrupt the blood supply in the brain.

We first imaged the *in vivo* dynamic change in blood oxygen saturation (${\mathrm{sO}}_{2}$) in a mouse brain using a deoxy-hemoglobin-dominated absorption wavelength of light at 620 nm. Oxygen challenges were performed to stimulate changes in the ${\mathrm{sO}}_{2}$ level in the mouse brain by manipulating the oxygen concentration of the mouse’s inhaled gas. In this study, a mixture of 95% oxygen and 5% nitrogen was initially used, with gaseous isoflurane for anesthesia. The mouse brain vasculature was first imaged through the intact skull in calibration mode. For the oxygen challenge, the mixture was changed to 5% oxygen and 95% nitrogen for 3 min; it was then changed back to the initial concentration to end the challenge.

To estimate the change in ${\mathrm{sO}}_{2}$ in a mouse brain using a single wavelength of light, a few assumptions are required. First, absorption in blood mainly comes from oxy- and deoxy-hemoglobin. Thus the absorption coefficient $\mu_{a}$ of blood can be calculated as

$$\mu_{a}=\ln(10)(\varepsilon_{{\mathrm{HbO}}_{2}}C_{{\mathrm{HbO}}_{2}}+\varepsilon_{\mathrm{Hb}}C_{\mathrm{Hb}}),$$ (5)

where ε is the molar absorption coefficient ($M^{-1}\;{\mathrm{cm}}^{-1}$), C is the concentration (M), and the subscripts ${\mathrm{HbO}}_{2}$ and Hb denote oxy- and deoxy-hemoglobin, respectively. The oxygen saturation in the blood is calculated as

$${\mathrm{sO}}_{2}=\frac{C_{{\mathrm{HbO}}_{2}}}{C_{{\mathrm{HbO}}_{2}}+C_{\mathrm{Hb}}}=1-\frac{C_{\mathrm{Hb}}}{C_{\mathrm{HbT}}},$$ (6)

where the total hemoglobin concentration ($C_{\mathrm{HBT}}$) is given by $C_{\mathrm{HBT}}=C_{{\mathrm{HbO}}_{2}}+C_{\mathrm{Hb}}$. Therefore, the change in the blood oxygen saturation can be calculated as

$${\Delta \mathrm{sO}}_{2}=-\frac{\Delta C_{\mathrm{Hb}}}{C_{\mathrm{HbT}}}.$$ (7)

Second, if we assume that the change in the total hemoglobin concentration in blood is insignificant, then a change in blood oxygen saturation signifies that $\Delta C_{\mathrm{Hb}}=-\Delta C_{{\mathrm{HbO}}_{2}}$. At a deoxy-hemoglobin dominant absorption wavelength, such as 620 nm, the ratio of $\varepsilon_{\mathrm{Hb}}/\varepsilon_{{\mathrm{HbO}}_{2}}$ is ∼7:1, thus a change in absorption is mainly due to a change in the concentration of deoxy-hemoglobin.^17^^,^^18^ Therefore, we can assume that $\Delta \mu_{a}\approx \ln(10)\varepsilon_{\mathrm{Hb}}\Delta C_{\mathrm{Hb}}$ and that the change in PA signal amplitude at 620 nm is proportional to the change in blood oxygen saturation.

To monitor the oxygen challenge, a tunable dye laser (CBR-D, Sirah GmbH), using DCM (SDL-550, Sirah GmbH) dissolved in ethanol as the gain medium, was pumped by the 532-nm pulsed laser (INNOSAB IS8II-E, Edgewave GmbH, 5-ns pulse width, 1-kHz pulse repetition rate) to generate laser light at 620 nm. The calibration (200×200  pixels, 30-min acquisition time) was performed at both 532 and 620 nm wavelengths, with an FOV of $3\times 3\;{\mathrm{mm}}^{2}$. We recorded the same FOV in widefield imaging mode at 620 nm during the oxygen challenge (Video S1) and calculated the signal differences pixel by pixel from the widefield images after temporal running averaging. Two oxygen challenge cycles were performed and analyzed [Fig. 3(a)]. The rate of signal change during the challenge was smaller than that during recovery from hypoxia [Fig. 3(b)], which is consistent with the results reported previously.^19^^,^^20^ To provide dual-wavelength measurements for ${\mathrm{sO}}_{2}$ calculation, widefield measurements at 532 nm were taken before and at 3 min into the oxygen challenge and reconstructed with the 532-nm calibration data. A vessel-segmentation and ${\mathrm{sO}}_{2}$ quantification algorithm was used to identify vessels and compute the ${\mathrm{sO}}_{2}$ within those vessels.^21^^,^^22^ The ${\mathrm{sO}}_{2}$ in the brain was found to have dropped significantly during the challenge [Fig. 3(c)].

**Fig. 3:**
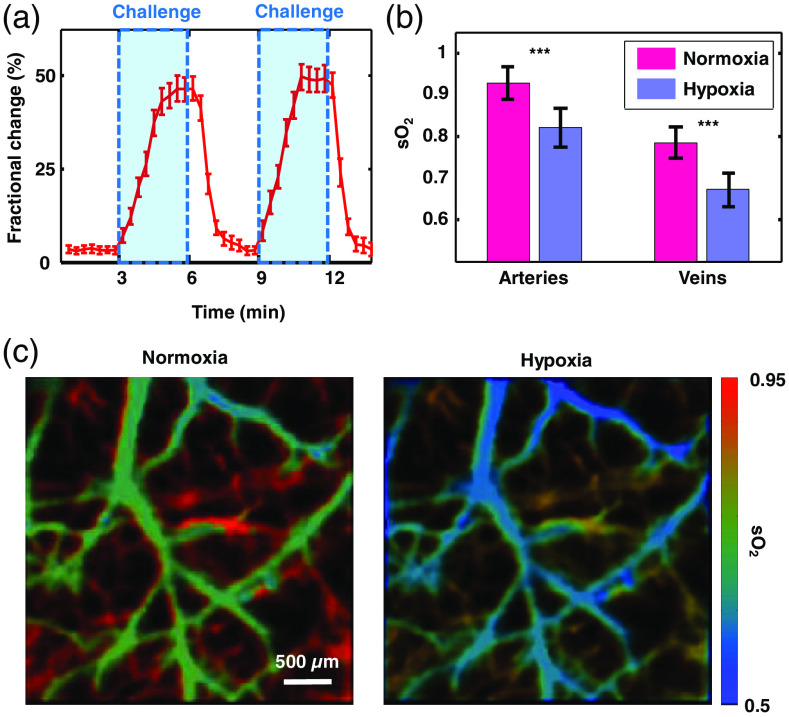
Change in blood oxygen saturation (${\mathrm{sO}}_{2}$) in a mouse brain due to oxygen challenge (n=4), imaged through an intact skull. (a) Time course of fractional change in signal amplitude with two cycles of oxygen challenge, measured at a deoxy-hemoglobin-dominant 620 nm wavelength. The imaging rate was 50  frames/s. The time window used for averaging was 20 s. Error bars, standard errors of the widefield measurements within averaging time windows. (b) Blood oxygen saturation in arteries and veins measured before (normoxia) and after (hypoxia) the challenge. Error bars, standard deviations. *** p<0.001, calculated by the two-sample t-test. (c) Blood oxygen saturation calculated before (left) and after (right) the challenge, using measurements acquired from two light wavelengths (532 and 620 nm) (Video S1, mp4, 8830 kB [URL: https://doi.org/10.1117/1.JBO.25.7.070501.1]).

In our second *in vivo* study, we demonstrated PATER’s ability to differentiate blood vessel patterns for potential biometric authentication applications. Biometric authentication utilizes unique biological characteristics of individuals to verify their identities. Internal characteristics such as vascular patterns can more securely identify an individual than external characteristics such as fingerprints^23^ because the internal characteristics are less exposed and contain *in vivo* physiological features—such as blood flow, arterial oxygenation, and venous oxygenation—that cannot be readily duplicated by others. Security applications based on internal biometric characteristics have great potential, but they require high processing speed and accuracy to be reliable.

First, one mouse was fixed in a stereotaxic frame and a region of the cortical vasculature was recorded in calibration mode [Fig. 4(a)]. Then, the same FOV was imaged in widefield imaging mode. During the widefield recording, we detached the mouse from the ER and then reattached it to the same position using a linear translational stage (PT1, Thorlabs, Inc.). Only noise was recorded while the mouse was detached, and signals were observed again when the mouse was reattached. This process was repeated for the second mouse. We then tried to reconstruct the widefield images of the first mouse’s vasculature using each of the two recorded calibration data sets. As a result, the widefield image reconstructed from the matched calibration data (the first mouse’s) revealed the original vasculature, whereas the widefield image reconstructed from the mismatched calibration data (the second mouse’s) could not [Fig. 4(b) and Video S2]. The correlation coefficients between the widefield reconstruction images and the calibration images were quantified [Fig. 4(c)]. The plot indicates that the widefield images reconstructed from the matched calibration data have a much higher correlation than those reconstructed from the mismatched calibration data. Also the vasculature is again recognizable after being detached and reattached to the ER. Several aspects of this proof-of-concept experiment still need to be addressed to make the technology more applicable. First, the region where the object is reattached to the ER needs to be the same as the calibrated region, requiring an effective repositioning method. Second, the deformation of soft tissue should be minimized for the current system, as it could change the boundary conditions. Third, the present PATER system requires calibration for each object; a universal calibration method is being explored, which will promise more biomedical applications in the future.

**Fig. 4:**
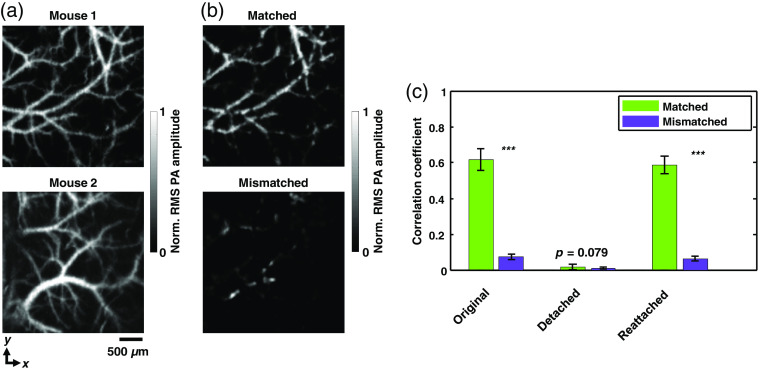
High-speed vascular recognition in mice through intact skulls (n=2). (a) RMS projections of brain vasculatures from mouse 1 and mouse 2, respectively, acquired in calibration mode. (b) Widefield images of mouse 1’s vasculature, reconstructed using the calibration data of mouse 1 (matched) and mouse 2 (mismatched), respectively. (c) Correlation between the widefield reconstructions and the RMS projections of mouse 1 and mouse 2, according to (b). The mouse was detached from and reattached to the ER to demonstrate the consistency of reconstruction. Error bars, standard deviations. ***, p<0.001, calculated by the two-sample t-test (Video S2, mp4, 718 kB [URL: https://doi.org/10.1117/1.JBO.25.7.070501.2]).

In summary, we have demonstrated PATER’s ability to quantify *in vivo* functional processes such as oxygen saturation changes in a mouse brain and also to identify vasculature patterns based on PATER’s unique detection method. PATER’s single-channel ultrasonic detection system can be a feasible alternative to a PACT’s multichannel ultrasound detection system. Compared to PACT, PATER has greatly reduced cost and system complexity, making it more affordable for portable applications, such as a wearable device to monitor vital signs in patients. Furthermore, since it can both identify vessel patterns and quantify functional processes, PATER can potentially provide comprehensive, secure, and robust biometric authentication.
